# Treatment adequacy and remission of depression and anxiety disorders and quality of life in primary care older adults

**DOI:** 10.1186/s12955-021-01851-4

**Published:** 2021-09-15

**Authors:** Catherine Lamoureux-Lamarche, Djamal Berbiche, Helen-Maria Vasiliadis

**Affiliations:** 1 Faculty of Medicine and Health Sciences, Campus de Longueuil – Université de Sherbrooke, 150 Place Charles-Le Moyne, Longueuil, QC J4K 0A8 Canada; 2 Centre de Recherche Charles-Le Moyne, 150 Place Charles Le Moyne, Longueuil, QC J4K 0A8 Canada

**Keywords:** Adequacy of care, Remission, Quality of life, Depression, Anxiety disorders, Older adults

## Abstract

**Background:**

Studies on the long-term outcomes of receiving adequate treatment for depression and anxiety disorders are scarce. The aims of this study were to assess the association between adequacy of care and remission of common mental disorders (CMD) and change in quality of life among a population of older adults consulting in primary care.

**Methods:**

The study was conducted among 225 older adults with a CMD who participated in the longitudinal ESA-Services study. Adequacy of care was assessed using administrative and self-reported data and was based on Canadian guidelines and relevant literature. CMD were measured at baseline and follow-up using self-reported measures (DSM-5 criteria) and physician diagnostic codes (International Classification of Diseases, 9^th^ and 10^th^ revisions) for depression and anxiety disorders. The remission of CMD was defined by the presence of at least one disorder at baseline and absence at follow-up. Quality of life was measured at baseline and follow-up using a visual analog scale and the Satisfaction With Life Scale. To estimate the probability to receive adequate/inadequate care, a propensity score was calculated, and analyses were weighted by the inverse probability. Weighted multivariable analyses were carried out to assess the remission of CMD and change in quality of life as a function of adequacy of care controlling for individual and health system factors.

**Results:**

Results showed that 40% of older adults received adequate care for CMD and 55% were in remission at follow-up. Adequacy of care was associated with remission of CMD (AOR: 0.66; CI 0.45–0.97; p-value: 0.032). Participants receiving adequate care had an improvement between baseline and follow-up of 0.7 (beta: 0.69, CI 0.18; 1.20, *p* = 0.008) point on the Satisfaction With Life Scale, while a marginal association was observed with improvement in HRQOL (beta: 2.83, CI 0.12; 5.79, *p* = 0.060).

**Conclusion:**

The findings contribute to the rare observational studies on the association between adequacy of care for CMD and long-term treatment effects. Future studies on population effectiveness should focus on patient indicators of quality of care which may better predict long-term outcomes for patients with depression and anxiety.

## Introduction

In Canada, predictions show that the number of older Canadians with mental disorders will double by 2041 [1]. The annual prevalence of common mental disorders (CMD) [2, 3] in community-dwelling older adults measured with structured interviews range between 1.1% to 11.6% for major depression [4–9] and between 5.6% to 17.2% for anxiety disorders [4–7, 9]. The chronic nature of CMD has also been reported. The 12-month persistence of major depression and anxiety disorders has been reported in 21% and 16% of older adults[10], with 3-year estimates of persistence reaching 25% and 30% [6].

While more than 70% of older adults consult a family doctor for their mental health symptoms [11], detection of CMD in primary care ranges between 24% and 64% [12–14]. Research has shown that over 50% of adult patients with depression or anxiety disorders do not receive guideline-concordant treatment [15–18]. Among older adults receiving antidepressant therapy, only 44% received an adequate dose and duration and had an appropriated number of physician follow-up visits [19].

Previous community-based studies showed that adults receiving guideline-concordant antidepressant therapies for major depression were more likely to be in remission at 3 and 6 months [20, 21]. Others have shown that treatment adequacy for anxiety disorders is associated with worse health-related quality of life at baseline (HRQOL) [22] but not with HRQOL improvement at 12 months [23].

To our knowledge, scarce are the studies that have documented the adequacy of care for CMD in older adults and even fewer have assessed the associations between adequacy of care and long-term outcomes among older adults and Canadian populations. The objectives of this study were to assess the association between adequacy of care and (1) remission of CMD and (2) change in HRQOL and satisfaction with life over a 3-year period among a population of community-dwelling older adults consulting in primary care. This longitudinal cohort study adds to the current literature by including a population of older adults covered under a public health plan for whom health survey and medico-administrative data on medical and pharmaceutical services used were available. This study design confers a number of advantages. First, it has been suggested that using either self-report or administrative data alone may underestimate the presence of mental disorders and therefore using information from both sources increases the validity of results [24, 25]. Second, this dataset allowed to control for a number of potential confounding individual and health system factors. Third, it allowed for a longer follow-up of treatment adequacy for depression and anxiety on study outcomes in a real-world setting.

## Method

The current study was conducted among 225 older adults aged 65 years and over with depression or an anxiety disorder participating in the 2011–2013 ‘’*Étude sur la Santé des Aînés-Services* (ESA-Services)’’ longitudinal study on aging and health service use with a follow-up at 3 years. The detailed methodology of the ESA-Services study and participants’ characteristics can be found elsewhere [26, 27]. Briefly, interviews at each wave were conducted face-to-face in the participant’s home by a trained interviewer using the structured computer assisted ESA-Services questionnaire which included questions on cognition, mental disorders and health status [9]. Older adults with moderate to severe cognitive decline, scoring lower than 22 at baseline on a French-Canadian translated and adapted version of the Mini-Mental State Examination for at home surveys in community living older adults [28, 29], were excluded from this study. The current study included participants who gave consent to access their medical administrative data during the study period and who were covered under the provincial public drug plan. Outpatient and inpatient healthcare consultations, physician diagnoses and pharmaceutical services delivered were obtained from the Régie de l’Assurance Maladie du Québec (RAMQ) and the Maintenance et exploitation des données pour l'étude de la clientèle hospitalière (MED-ECHO) provincial databases. The longitudinal ESA-Services study was approved by the research ethics boards of the *CISSS-Montérégie-Centre* and the *CIUSSS-de l’Estrie-Centre Hospitalier Universitaire de Sherbrooke*. Quebec’s *Commission d’accès à l’information* gave approval for the linkage of individual health survey and administrative data.

### Measures

#### Common mental disorders (CMD)

The presence of a CMD was based on self-reported health survey information and the presence of a physician diagnosis for depression or an anxiety disorder. The past 6-month self-reported presence of major depression, specific and social phobia, agoraphobia, panic disorder, generalized anxiety disorder, at baseline and 3-year follow-up, was based on DSM-5 criteria [30] with the exception of  the presence of impairment in at least one area of functioning (criteria B). The presence of a physician diagnosis of a CMD was based on information reported in provincial health administrative databases in the 6 months prior and following the baseline and follow-up interviews. The following International Classification of Disease (ICD-9, ICD-10) diagnostic codes were considered for depression: 311.0; 311.9; 300.4; F32; F33; F34.1 [31–35]. The codes 300.0, 300.2, F40 and F41 were included for anxiety disorders [34, 35].

### Three-year remission of CMD

Older adults with depression or an anxiety disorder at baseline who did not meet the criteria for a DSM-5 disorder [30] at follow-up and did not have a physician diagnosis in the 6 months prior and after the follow-up interview were categorized as being in remission. The persistence of CMD was defined by the presence of at least one disorder (self-reported or diagnosed) at baseline and follow-up.

### Quality of life

#### Health-related quality of life

HRQOL was assessed at both baseline and follow-up using a visual analog scale where participants were asked “Indicate where your health state is today on a scale of 0 to 100, with 0 representing the worst health state imaginable and 100 representing the best health state imaginable." The use of visual analog scales has previously been used to ascertain HRQOL among adults with depression and anxiety disorders [36, 37] and older adult populations [37, 38].

#### Life satisfaction

Life satisfaction was measured using an adapted version of the French validated Satisfaction With Life Scale (SWLS) [39, 40]. The adapted SWLS includes 5 questions on a 5-point Likert Scale ranging from ‘’completely agree’’ to ‘’completely disagree’’. The version used in the ESA-Services questionnaire was adapted for older adults by modifying the original 7-point Likert scale to a 5-point Likert scale to decrease the burden put on participants. The original French version showed good reliability and validity in older adults [40]. Satisfaction with life was assessed at baseline and follow-up.

### Minimally adequate treatment

Minimally adequate treatment for CMD was based on Canadian clinical guidelines for adults and older adults and published reports. Minimal indicators of adequate treatment for depression and anxiety disorders in an epidemiologic context have previously been used in population-based studies in Canada and elsewhere [3, 15, 18]. For depression, minimally adequate treatment was defined by the presence of at least one prescription for an antidepressant medication and 4 medical visits with a general practitioner (GP) within 3 months to adjust the medication dosage and to monitor side effects or 8 psychotherapy visits, group or individual therapy sessions in the 12 months following the index date [41–44]. For anxiety disorders, older adults had to receive at least one prescription for an antidepressant, anxiolytic or another recommended drug with 4 follow-up visits with a GP within 3 months or 7 psychotherapy consultations, group or individual therapy sessions within 12 months of the index date [15, 45, 46]. Other medication recommended for the treatment of anxiety disorders included selected atypical antipsychotic, anticonvulsant and other drug such as buspirone, atomoxetine and selegiline [45]. The previous definition was used for all anxiety disorders with the exception of specific phobia, which was defined by the presence of 5 psychotherapy or therapy consultations in the 12 months following the index date as pharmacotherapy treatment is not recommended for this disorder [45, 47]. Beers criteria regarding the use of benzodiazepines in older adults have evolved over time with limited restrictions of use in 2012 [48]. The recent criteria highlighted that benzodiazepines may be appropriate for the treatment of severe generalized anxiety disorder [49]. Limited clinical guidelines are available for anxiety disorders in older adults and therefore from an epidemiologic perspective, where data on patient health and treatment history and preference are not available, we included the presence of a benzodiazepine prescription in the definition of adequacy of care in addition to the presence of 4 primary care visits allowing for a close follow-up of patients. For older adults with a self-reported CMD, the index date was identified as the first prescription or therapy visit in the 6 months prior and following the first interview. The index date for participants with a diagnostic was defined as the date of the first diagnostic in the same 12-month timeframe (6 months prior and after the first interview).

Participants were also asked in the ESA-Services questionnaire how many visits they had with a psychologist in the last 6 months. Psychotherapy or therapy consultations captured in the administrative database were compared to self-reported consultations to avoid double counting.

Using these criteria, 14 categories were created to capture adequacy according to the different data sources and are as follows: 1) self-reported depression, pharmacotherapy with antidepressants; 2) self-reported depression, psychotherapy or therapy with physician; 3) diagnosed depression, pharmacotherapy with antidepressants; 4) diagnosed depression, psychotherapy or therapy with physician; 5) self-reported or diagnosed depression, self-reported psychotherapy; 6) self-reported anxiety disorder, pharmacotherapy with antidepressants; 7) self-reported anxiety disorder, pharmacotherapy with anxiolytics; 8) self-reported anxiety disorder, pharmacotherapy using other medications; 9) self-reported anxiety disorder, psychotherapy or therapy with physician; 10) diagnosed anxiety disorder, pharmacotherapy with antidepressants; 11) diagnosed anxiety disorder, pharmacotherapy with anxiolytics; 12) diagnosed anxiety disorder, pharmacotherapy with other medications; 13) diagnosed anxiety disorder, psychotherapy or therapy with physician; 14) self-reported or diagnosed anxiety disorder, self-reported psychotherapy.

Participants in each of these categories were examined individually and further defined as receiving adequate or inadequate care. Participants with a CMD who did not meet the criteria for adequate care for one definition were categorized as receiving inadequate care.

### Other individual and health system variables

Other variables considered in the analyses were based on Andersen’s behavioral model on healthcare service utilization, where individual factors influence health and help-seeking behaviors and patient outcomes [50]. Individual predisposing variables measured at baseline included: sex (male/female), age, marital status (married/not married) and education (0–7 years/8 years and over). Social and material deprivation indexes and social support were considered as potentially enabling individual factors. The Pampalon deprivation indexes were used to assess the social and material deprivation of the area of residence [51]. These indexes were calculated from previous Canadian census and were based on socioeconomic geographic indicators. Social and material indexes ranged from 1 (lowest deprivation) to 5 (highest deprivation). Social support was measured at baseline using three questions in the ESA-Services questionnaire referring to instrumental and emotional support with scores ranging between 0 to 3.

We included the following individual need factors measured at baseline: cognitive functioning, number of chronic diseases, severity of depression and anxiety symptoms, number of daily hassles and the presence of an inpatient stay in the 3 years following the baseline interview. Possible cognitive functioning scores ranged from 22 to 30 with higher scores indicating better cognition [29]. The number of chronic diseases was assessed using a list of 17 physical conditions. The severity of depressive symptoms was assessed using the 10-item Kessler Psychological Distress Scale (K10) [52] with scores ranging between 10 and 50. The severity of symptoms of anxiety was measured using the 7-item Generalized Anxiety Disorder (GAD-7) questionnaire [53] with scores ranging between 0 and 21. For both instruments, higher scores represent higher severity of symptoms. A reduced French version of the Hassles Scale [54] was used to assess the number of daily hassles.

In the current study, health care system enabling factors such as retention and attraction indexes for primary care and psychiatric services and the type of clinics where participants were recruited were also considered. Retention indexes represent the proportion of primary care and psychiatric services used locally by the population of a territory over all services dispensed to this population regardless of the territory. Attraction indexes measure the proportion of primary care and psychiatric services used locally by the population of a territory over all services dispensed on the territory [55]. The type of primary care clinics where participants were recruited at baseline was defined as small primary care clinics (private clinics < 3 GP) and large primary care clinics (private clinics ≥ 3 GP, local community service centers, family medicine groups).

### Analysis

Among the 225 older adults, 221, 224 and 218 participants had available data for remission of CMD, change in HRQOL and life satisfaction respectively. A total of 49 and 37 participants had missing items on the K10 or the GAD-7 and data for both these variables were imputed. For participants who had answered at least 6 out of the 10 items on the K10, the missing items were replaced with the average score of the items that were completed. Similarly, for the GAD-7, the average score of the items answered was used to replace the missing items for participants who responded to at least 4 out of the 7 items. Older adults who had at least 5 unanswered items on the K10 or at least 4 unanswered items on the GAD-7 and for whom a score was available at follow-up, this last score was used. A total of 23 older adults had missing data on the deprivation indexes and data were imputed considering age and sex based on a previously published technique for categorical variables [56].

The distribution and homogeneity of residuals of the change in quality of life variables were assessed. Although the distribution of residuals was symmetrical and seemed to follow a normal distribution graphically, the Kolmogorov–Smirnov and Shapiro–Wilk tests showed significant results indicating that the postulate was not respected. Generalized linear models (GLM) were therefore conducted, which tend to be more robust when the distribution of errors is not normal.

Bivariate logistic and GLM were used to assess the association between remission of CMD and change in quality of life as a function of adequacy of treatment and potential confounding factors. All potential confounding factors that were associated with remission of CMD and change in quality of life with a significant level of 0.10 were used to create a propensity score to calculate the probability of receipt of adequate and inadequate treatment for CMD. This technique has previously been used to account for the potential confounding effects of variables associated with treatment received in real-world conditions [57].

Multivariable logistic analyses were carried out to assess the association between adequacy of care and remission of CMD using a significance level of 0.05. Adjusted odds ratios (AOR) with their 95% confidence intervals (CI) and p values are reported. Multivariable GLM were conducted to evaluate the association between adequacy of care and change in HRQOL and life satisfaction during follow-up. A significant statistical threshold of 0.05 was used and beta estimates representing the change between baseline (T1) and follow-up (T2) (T2-T1) are reported with their 95% CI and corresponding p values. All multivariable models were weighted by the inverse probability of receiving adequate/inadequate care and were controlled for study variables used to create the propensity score as well as sex and age. All analyses were performed with SPSS 27.0, while the propensity score was estimated using SAS 9.4.

Given the changes in the recommendations for benzodiazepine use in older adults in the last decade, additional analyses were conducted to assess the association between study outcomes and treatment adequacy of CMD, defined without considering the presence of a benzodiazepine prescription. Our hypothesis was that older adults using benzodiazepines would reflect different clinical profiles and health outcomes.

## Results

At baseline, 27 (12.0%) and 174 (77.3%) older adults had depression and an anxiety disorder only and 24 (10.7%) had both. Overall, 55.2% of older adults with a CMD were in remission while 21.6% and 42.6% of older adults with depression and anxiety were persistent at follow-up. Criteria for treatment adequacy was filled in 40.0%, 56.9% and 37.9% of older adults with a CMD overall, depression and anxiety disorders, respectively.

The bivariate associations between adequacy of mental health care and remission of CMD and change in quality of life with a threshold of 0.10 are presented in Tables 1 and 2.

**Table 1:** Study sample characteristics as a function of the presence of remission of depression and anxiety disorders at follow-up

	Overall sample (n = 225)*	Yes (n = 122)*	No (n = 99)*	Odds ratio (95% CI)	p value
	n (%)				
*Individual factors*					
Adequacy of care for CMD					
Yes	90 (40)	39 (32)	48 (48)	**0.50 (0.29–0.86)**	**0.013**
No	135 (60)	83 (68)	51 (52)	REF	
Sex					
Female	152 (68)	78 (64)	70 (71)	0.73 (0.42–1.30)	0.288
Male	73 (32)	44 (36)	29 (29)	REF	
Marital status					
Married	128 (57)	76 (62)	52 (53)	1.49 (0.87–2.56)	0.144
Not married	97 (43)	46 (38)	47 (47)	REF	
Education					
Primary	49 (22)	29 (24)	19 (19)	1.33 (0.69–2.55)	0.394
Secondary/post-secondary/university	175 (78)	92 (76)	80 (81)	REF	
Social deprivation index					
1	33 (15)	20 (17)	13 (13)	1.99 (0.83–4.78)	0.125
2	39 (18)	22 (19)	17 (17)	1.67 (0.73–3.82)	0.224
3	42 (19)	23 (19)	18 (19)	1.65 (0.73–3.73)	0.228
4	47 (21)	29 (25)	18 (19)	2.08 (0.94–4.60)	0.070
5	58 (27)	24 (20)	31 (32)	REF	
Material deprivation index					
1	43 (20)	22 (19)	20 (21)	1.38 (0.59–3.20)	0.460
2	48 (22)	24 (20)	23 (24)	1.30 (0.57–2.96)	0.526
3	41 (19)	22 (19)	19 (19)	1.45 (0.62–3.39)	0.394
4	42 (19)	30 (25)	10 (10)	**3.75 (1.49–9.47)**	**0.005**
5	45 (20)	20 (17)	25 (26)	REF	
Hospitalisation					
Yes	54 (24)	25 (20)	28 (28)	0.65 (0.35–1.22)	0.179
No	171 (76)	97 (80)	71 (72)	REF	
	*Means (SD)*	*Means (SD)*	*Means (SD)*		
Age	73.15 (5.37)	72.97 (5.46)	73.39 (5.30)	0.99 (0.94–1.04)	0.569
Cognitive functioning	28.34 (1.69)	28.35 (1.70)	28.35 (1.71)	1.00 (0.86–1.17)	0.996
Number of chronic disorders	4.41 (2.39)	4.34 (2.44)	4.53 (2.31)	0.97 (0.87–1.08)	0.556
Psychological distress	20.67 (6.85)	19.38 (5.95)	22.33 (7.58)	**0.94 (0.90–0.98)**	**0.002**
Anxiety symptom severity	5.59 (3.53)	5.12 (3.14)	6.21 (3.88)	**0.91 (0.84–0.99)**	**0.025**
Number of daily hassles	10.30 (6.86)	10.29 (6.91)	10.41 (6.92)	1.00 (0.96–1.04)	0.891
Social support index	2.77 (0.57)	2.78 (0.58)	2.75 (0.56)	1.10 (0.69–1.75)	0.686
Health system factors					
Type of primary care practices	n (%)	n (%)	n (%)		
Small (< 3 GP)	93 (43)	43 (37)	49 (50)	0.59 (0.34–1.02)	0.058
Large clinics (≥ 3 GP)	125 (57)	73 (63)	49 (50)		
	*Mean (SD)*	*Mean (SD)*	*Mean (SD)*		
Retention index – GP	0.64 (0.20)	0.66 (0.18)	0.63 (0.21)	2.21 (0.57–8.60)	0.255
Retention index – Psychiatrist	0.31 (0.35)	0.32 (0.35)	0.31 (0.35)	1.14 (0.54–2.44)	0.729
Attraction index – GP	50.83 (32.74)	51.29 (31.36)	49.68 (33.96)	1.00 (0.99–1.01)	0.714
Attraction index – Psychiatrist	5.79 (8.50)	5.68 (8.34)	5.71 (8.42)	1.00 (0.97–1.03)	0.978

Significant results (p < 0.05) are in bold

*A total of 221 participants had available data for remission of CMD. There is missing data for the following variables: education (1), deprivation indexes (6), psychological distress (1), anxiety symptoms (2), type of primary care clinics (7), attraction index – psychiatrist (4)

CI: Confident Intervals; CMD: Common mental disorders; GP: General practitioner; SD: Standard deviation

**Table 2:** Bivariate associations between study variables and change in health-related quality of life and satisfaction with life

	Change in HRQOL between T1 and T2 n = 224*		Change in satisfaction with life between T1 and T2 n = 218*	
	Unadjusted Beta estimates (95% CI)	p value	Unadjusted Beta estimates (95% CI)	p value
Adequacy of care for CMD				
Yes vs no	4.48 (− 0.71; 9.66)	0.091	0.86 (− 0.04; 1.75)	0.060
Sex				
Female vs male	2.01 (− 3.44; 7.45)	0.470	0.78 (− 0.16; 1.72)	0.104
Age	− 0.11 (− 0.58; 0.37)	0.666	0.04 (− 0.05; 0.12)	0.387
Marital status				
Married vs not married	0.98 (− 4.17; 6.13)	0.708	− 0.03 (− 0.92; 0.87)	0.956
Education				
Primary vs secondary/post-secondary/university	− 4.10 (− 10.30; 2.09)	0.194	0.39 (− 0.68; 1.46)	0.471
Social deprivation index				
1	0.41 (− 7.73; 8.55)	0.922	− 0.54 (− 1.97; 0.90)	0.466
2	− 6.31 (− 14.04; 1.42)	0.110	− 0.53 (− 1.91; 0.84)	0.447
3	− 2.31 (− 9.87; 5.26)	0.550	− 0.42 (− 1.76; 0.93)	0.544
4	6.55 (− 0.83; 13.92)	0.082	− 0.07 (− 1.38; 1.25)	0.923
5	REF		REF	
Material deprivation index				
1	− 0.25 (− 8.33; 7.83)	0.952	0.10 (− 1.33; 1.52)	0.896
2	− 3.25 (− 11.11; 4.61)	0.418	− 0.89 (− 2.28; 0.50)	0.210
3	1.56 (− 6.68; 9.79)	0.711	− 0.46 (− 1.90; 0.97)	0.526
4	− 5.50 (− 13.63; 2.63)	0.185	− 0.42 (− 1.85; 1.02)	0.571
5	REF		REF	
Cognitive functioning	− 1.02 (− 2.53; 0.48)	0.183	− 0.04 (− 0.31; 0.22)	0.746
Number of chronic disorders	0.62 (− 0.44; 1.69)	0.250	− 0.03 (− 0.21; 0.16)	0.759
Psychological distress	0.27 (− 0.11; 0.65)	0.158	0.02 (− 0.04; 0.09)	0.463
Anxiety symptom severity	0.55 (− 0.18; 1.27)	0.143	− 0.002 (− 0.13; 0.13)	0.980
Number of daily hassles	**0.39 (0.02; 0.76)**	**0.038**	0.04 (− 0.28; 0.10)	0.275
Social support index	0.05 (− 4.46; 4.55)	0.983	− 0.06 (− 0.93; 0.81)	0.895
Hospitalisation				
Yes vs no	− 2.54 (− 8.50; 3.42)	0.403	− 0.58 (− 1.63; 0.46)	0.272
Type of primary care practices				
Small (< 3 GP) vs large clinics (≥ 3 GP)	− **5.94 (**− **11.17; 0.70)**	**0.026**	0.51 (− 0.38; 1.41)	0.260
Retention index – GP	**13.05 (0.11; 26.00)**	**0.048**	− 0.71 (− 2.97; 1.56)	0.540
Retention index – Psychiatrist	6.01 (− 1.25; 13.28)	0.105	− 0.62 (− 1.89; 0.66)	0.342
Attraction index – GP	0.03 (− 0.05; 0.11)	0.433	− 0.01 (− 0.02–0.01)	0.432
Attraction index – Psychiatrist	0.18 (− 0.13; 0.48)	0.257	− 0.01 (− 0.07–0.04)	0.624

Significant results (p < 0.05) are in bold

*A total of 224 and 218 participants had available data on the visual analog scale and the Satisfaction With Life Scale. There is missing data for the following variables: education (1), deprivation indexes (6), psychological distress (1), anxiety symptoms (2), type of primary care clinics (7), attraction index – psychiatrist (4)

CI: Confident Intervals; CMD: Common mental disorders; GP: General practitioner; HRQOL: Health-related quality of life

After controlling for potential confounders, receipt of adequate care was associated with a decreased likelihood of remission (AOR: 0.66; CI 0.45–0.97; p-value: 0.032) (Table 3). Multivariable models also showed that receipt of adequate mental health care was associated with an average improvement of 0.7 points in satisfaction with life (beta: 0.69, CI 0.18; 1.20, *p* = 0.008) after controlling for the baseline score, while a marginal association was observed with improvement in HRQOL (beta: 2.83, CI 0.12; 5.79, *p* = 0.060) (Table 3).

**Table 3:** Multivariate associations between adequacy of care and remission of CMD and change in quality of life

	Remission of CMD N = 206*		Change in HRQOL between T1 and T2 n = 209*		Change in satisfaction with life between T1 and T2 n = 204*	
	Weighted adjusted odds ratio** (95% CI)	p value	Weighted adjusted Beta estimates** (95% CI)	p value	Weighted adjusted Beta estimates** (95% CI)	p value
Adequacy of care for CMD						
Yes vs no	**0.66 (0.45–0.97)**	**0.032**	2.83 (− 0.12; 5.79)	0.060	**0.69 (0.18; 1.20)**	**0.008**
Sex						
Female vs male	0.86 (0.61–1.23)	0.415	1.22 (− 1.48; 3.92)	0.377	0.75 (0.28–1.21)	**0.002**
Age	**0.95 (0.92–0.98)**	**0.003**	− **0.35 (**− **0.60; **− **0.09)**	**0.007**	0.02 (− 0.02; 0.07)	0.305
Social deprivation index	0.91 (0.81–2.22)***	0.143	− 0.18 (− 1.13; 0.78)***	0.720	− 0.17 (− 0.33; 0.001)***	0.051
Material deprivation index	1.16 (1.03–1.32)***	**0.016**	− 0.79 (− 1.74; 0.16)***	0.104	0.09 (− 0.07; 0.26)***	0.263
Psychological distress	**0.89 (0.84–0.95)**	** < 0.001**	− 1.20 (− 1.67; − 0.72)	** < 0.001**	− 0.01 (− 0.10; 0.07)	0.769
Anxiety symptom severity	**1.14 (1.01–1.28)**	**0.029**	2.04 (1.14; 2.94)	** < 0.001**	− 0.12 (− 0.28; 0.03)	0.119
Number of daily hassles	0.98 (0.96–1.00)	0.090	0.13 (− 0.05; 0.31)	0.143	0.003 (− 0.03; 0.03)	0.852
HRQOL at baseline			− **0.69 (**− **0.77; **− **0.61)**	** < 0.001**		
Life satisfaction at baseline					− **0.35 (**− **0.41; **− **0.28)**	** < 0.001**
Type of primary care practices						
Small (< 3 GP) vs large clinics (≥ 3 GP)	0.37 (0.25–0.55)	** < 0.001**	**3.03 (0.09; 5.98)**	**0.043**	− 0.13 (− 0.66; 0.39)	0.617
Retention index – GP	0.79 (0.29–2.11)	0.634	**12.60 (5.11; 20.09)**	** < 0.001**	− 0.58 (− 1.92; 0.76)	0.396

Significant results (p < 0.05) are in bold

*Number of participants included in multivariable models

**Variables considered to create the propensity score: deprivation indexes, psychological distress, anxiety symptoms, number of daily hassles, type of primary care practices and retention index for general services

**Deprivation indexes were included as continuous variables in multivariable models

CI: Confident Intervals; CMD: Common mental disorders; GP: General practitioner; HRQOL: Health-related quality of life

Sensitivity analyses (not presented) showed that when the presence of a benzodiazepine prescription was removed from the definition of adequate care, receipt of adequate  care for CMD decreased to 29.8%. Receipt of adequate care remained associated with remission (AOR: 0.58, CI 0.38–0.87, *p* = 0.009), while the associations with change in HRQOL (beta: 2.68, CI − 0.50; 5.86, *p* = 0.098) and satisfaction with life (beta: 0.22, CI − 0.32; 0. 77, *p* = 0.424) were no longer significant.

## Discussion

The objectives of this study were to assess the effect of receipt of adequate care on the remission of CMD and change in quality of life in older adults. The current findings showed that 57% of participants with depression and 38% with an anxiety disorder received an appropriate treatment according to guidelines. Previous population-based studies conducted in adult populations in Canada showed similar results regarding adequacy of care for depression. One of these studies was conducted among a nationally representative Canadian population of adults aged 15 years and over and the other included a population of adults in Quebec who were consulting a general practitioner [18, 58]. In a primary care study in the Netherlands, Smolders and colleagues [16] reported that close to 42% of adult patients received guideline-concordant care for depression, which is lower than what is observed in the current study. However, the authors did not used minimal indicators of receipt of adequate care, which may partly explain the difference observed. Our study also showed that 38% of older adults with an anxiety disorder received an appropriate treatment, which is higher than previous studies. Roberge and colleagues [15] reported in a Canadian sample of adults with anxiety disorders that only 21% received minimally adequate treatment. When looking at adults who had at least one consultation with a physician, this percentage reached 56% [15]. Other studies in the Netherlands and the United States reported that close to 28% of adults consulting in primary care received an adequate treatment for anxiety [16, 17]. The majority of these studies evaluated the presence of anxiety disorders based on validated structured interviews [15, 17] and did not include physician diagnoses. In our study, both clinical diagnoses and self-reported anxiety disorders based on DSM-5 criteria were considered.

Close to 55% of older adults were in remission 3 years later. When looking at persistence, the findings showed that 22% and 43% of participants with depression and anxiety were persistent at follow-up. A previous population-based study conducted among a representative sample of older adults reported similar results for the persistence of depression [6], but slightly lower estimates for anxiety disorders at 30% persistence 3 years later. The results observed for persistence of depression may also reflect the estimated prevalence of treatment-resistant depression in primary care settings in Canada [59]. This difference may in part be attributed to the definition used. In the current study, physician diagnoses were considered, and the definition of self-reported depression and anxiety did not include DSM-5 criteria regarding impairment in functioning. The study sample also included a population of older adults who consulted in primary care as opposed to general population samples [6] and may reflect a sample with more severe and chronic conditions. Further, the majority had consulted their regular physician during that study period which may reflect closer follow-up of care.

Participants who received an adequate treatment were less likely to be in remission after 3 years of follow-up. This does not concord with previous observation studies conducted among 165 adults consulting in mental health clinics for depression reporting those who received adequate treatment were 2 to 3 times more likely to be in remission at 3 and 6 months of follow up [20, 21]. The divergent results may be due to the fact that in these two latter studies, samples included younger adults followed in specialized clinics and the follow-up was significantly shorter. Hepner and colleagues [60] reported results from three randomized controlled trials (RCT) conducted in primary care settings and showed that an increase in quality of depression care was associated with a lower risk of persistence at 18 and 24 months. One of the rare studies among adults with anxiety was conducted among the control group of a RCT and showed that participants with a panic disorder receiving adequate treatment were more likely to be in remission at 6 and 12 months [22]. The follow-up periods in these studies may not have been long enough to see the natural course of an episode of depression or an anxiety disorder. As shown in a previous longitudinal study conducted among a representative sample of community living older adults, mood and anxiety disorders last on average up to 23.4 months [10]. Overall, only two observational studies were found, and these studies were conducted among a specific population of adults with major depression consulting in mental health clinics [20, 21]. Further, the number of studies that included anxiety disorders is very limited. In our study, patients with only anxiety represented 77% of the overall sample, which makes it difficult to compare our results with other studies. Additional analyses showed that older adults having a physician diagnosis of a CMD at baseline were 2.5 times more likely to receive an adequate treatment and to be persistent at follow-up compared to participants with a self-reported CMD only. From a health system perspective, this is encouraging because these patients are more likely to be detected and to receive adequate care. This may reflect that diagnosed cases may have more complex clinical profiles necessitating closer follow-up of care.

The current study also reported on the association between adequacy of care received for depression and anxiety disorders and changes in HRQOL and satisfaction with life over a 3-year period. Adequacy of care for CMD was associated with a change in satisfaction with life, while a marginal association was observed for change in HRQOL. When looking at the association between adequacy of care and change in quality of life in individuals with depression and anxiety disorders separately, we observed that adequacy of care for depression was not associated with change in either HRQOL and satisfaction with life, while receiving an adequate treatment for anxiety disorders was associated with improvement in HRQOL and satisfaction with life. Similarly to the current results, a secondary analysis of data from a RCT on the treatment of depression with antidepressants in the United Stated among adults aged 18 years and older showed that adequacy of depression care was not associated with scores on the physical and mental health dimensions of the Medical Outcomes Study 36-Item Short Form Health Survey (SF-36) at 1, 3 and 6 months and this after controlling for the score at baseline [61]. However, a population-based study reported in a sample of 139 adults recruited in a mental health community clinic [23] in the Netherlands that care following clinical guidelines for anxiety disorders was not associated with a 12-month change in quality of life measured using the World Health Organization Quality of Life Questionnaire [23]. Despite the fact that receiving adequate care for CMD was marginally associated with change in HRQOL, no study has reported on minimally clinically important difference for mental disorders. Others have estimated between 5 to 12 points on the visual analog scale in populations with specific chronic physical diseases [62–64]. Future studies should focus on different patient indicators of quality of care which may better predict clinically significant change in quality of life scores in people with depression and anxiety disorders.

Overall, our results do not concord with previous studies that assessed the association between adequacy of care and remission of CMD and quality of life. Previous research was mainly conducted in controlled conditions with short-term follow-ups. Clinical guidelines used to define adequate care are mostly based on systematic reviews and meta-analyses of RCTs [65]. However, experts have highlighted the important gap between treatment outcomes observed in RCTs and those from real-world conditions [65–67]. A recent editorial stated that the majority of patients with mental disorders seen in clinical practices have comorbidities, which is usually an exclusion criterion in RCTs [68]. Further, due to their age, older adults are often excluded from RCTs, which represents real clinical challenges for treating aging populations worldwide [69]. As recently highlighted by Leichsenring et al. [68], more pragmatic design studies and long-term RCTs that include important outcomes such as quality of life, disability, utility values and costs are needed to assess the real-world effectiveness of mental health treatments and interventions.

Sensitivity analyses excluding the use of benzodiazepines in the definition of adequacy of care showed similar results on remission, while the associations with change in satisfaction with life was no longer significant. Additional analyses also showed that participants who had a benzodiazepine prescription in the 3 months following the index date were more likely to be persistent, to receive adequate care and to have more severe symptoms. The association with change in quality of life was not significant. These results may reflect a more complex clinical profile and closer follow-up. Future studies should identify which components or combination of treatment for CMD lead to better long-term outcomes in older adults.

The current study had a number of strengths and limitations. To our knowledge, no study so far has assessed the association between adequacy of care and remission of CMD and change in quality of life in a population of older adults consulting in primary care practices and this in a Canadian public health care context. The longitudinal study design combined health survey and administrative data. Both self-reported and administrative data were used to assess the remission of CMD and adequacy of care. Using both sources of data to identify the presence/absence of depression and anxiety disorders minimised the potential recall and social-desirability bias. Schülssler-Fiorenza Rose et colleagues [24] highlighted the importance of combining self-reported and administrative data, particularly for diseases with diagnostic codes with low sensitivity like depression. A Canadian case-study similarly highlighted the difficulties in measuring quality of care for depression using administrative database only [70]. Further, the current study also addressed limitations in previous studies where both pharmacological and psychological treatments were considered in the definition of adequacy of care and this using self-reported and administrative data which also included consultations with psychologists in the private sector and not covered under the public health plan. Finally, we used propensity analyses to account for the potential confounding effect of factors associated with the treatment received in real-world settings.

There are also a number of limitations that need to be considered. First, participants who accepted to participate to the ESA-Services study may differ from those who did not participate. Available data however showed that they were similar with regards to sex, psychological distress and anxiety symptoms. Also, older adults who completed the second interview and those lost at follow-up may also differ, however, analyses showed that older adults who participated at follow-up were similar to those lost to follow-up regarding adequacy of depression, anxiety disorders and CMD. Second, the results may be subject to information bias. Classification errors in the diagnostic codes are possible since physicians are not required to report diagnoses to be paid for consultations. To minimize the influence of this bias, both self-reported and administrative data were used to measure the presence/absence of CMD. The RAMQ pharmaceutical administrative database provides information on all medications delivered on an outpatient basis and does not allow to evaluate if the medication was consumed. Since CMD were measured during face-to-face interviews, there is also the possibility of social desirability bias. Interviewers were trained to use the structured questionnaire and invited participants to carry out the interview in the most isolated area of the home if there was another person present. Third, adequacy of care was based on minimally adequate treatment definitions previously published in population-based studies [15, 18] as it is not possible to account for the clinical profile, history of treatment and treatment preference of each patient when adequacy of care is measured at the population level [18]. All medication classes recommended in clinical guidelines and the number of psychotherapy visits, group and individual therapy sessions, were included in the definition. Also, psychological visits in the private sector may not have been captured for all participants as psychological visits were only measured in the 6 months before the first interview which may have underestimated the number of people being categorized as having received adequate care. Finally, the results of this study could not be generalized to all older populations as this study was conducted among a convenience sample of older adults with a CMD living at home and consulting in primary care practices.

## Conclusion

The current findings contribute to the rare observational studies on the association between adequacy of care for depression and anxiety disorders and long-term effects of treatment in an epidemiologic context. In our study, adequacy of care was associated with remission of CMD and change in satisfaction with life. Future studies on population effectiveness should focus on different patient indicators of quality of care which may better predict clinically significant long-term outcomes of treatment for people with depression and anxiety.

## Data Availability

The authors are not legally authorised to share or publicly publish linked survey and health administrative data due to privacy or ethical restrictions related to the use of administrative provincial health data. Participants were not requested to give informed consent for data sharing. The province of Québec's 'Commission d'Accès à l'Information' gave approval to merge these datasets. Requests for access to the data should be addressed to the research ethics board of the *CIUSSS de l’Estrie-Centre Hospitalier Universitaire de Sherbrooke*.

## Abbreviations

AOR: Adjusted odds ratio
CI: Confidence interval
CMD: Common mental disorders
ESA-Services: Étude sur la Santé des Aînés-Services
ICD: International classification of diseases
GAD-7: 7-Item Generalized Anxiety Disorder
GP: General practitioner
HRQOL: Health-related quality of life
K10: 10-Item Kessler Psychological Distress Scale
MED-ECHO: Maintenance et exploitation des données pour l'étude de la clientèle hospitalière
RAMQ: Régie de l’Assurance Maladie du Québec
RCT: Randomized controlled trial
SWLS: Satisfaction With Life Scale
