# Correction to: Treatment adequacy and remission of depression and anxiety disorders and quality of life in primary care older adults

**DOI:** 10.1186/s12955-021-01870-1

**Published:** 2021-09-30

**Authors:** Catherine Lamoureux‑Lamarche, Djamal Berbiche, Helen‑Maria Vasiliadis

**Affiliations:** 1 Faculty of Medicine and Health Sciences, Campus de Longueuil – Université de Sherbrooke, 150 Place Charles‑Le Moyne, Longueuil, QC J4K 0A8 Canada; 2 Centre de Recherche Charles-Le Moyne, 150 Place Charles Le Moyne, Longueuil, QC J4K 0A8 Canada

## Correction to: Health Qual Life Outcomes (2021) 19:218 https://doi.org/10.1186/s12955-021-01851-4

The authors found two minor errors in the text, abstract and Table 2:

1. In the abstract and results section, “(beta: 2.83, CI 0.12; 5.79, *p* = 0.060)” should instead read “(beta: 2.83, CI − 0.12; 5.79, *p* = 0.060)”.
2. In Table 2 (change in HRQOL), the unadjusted beta estimate and confidence interval for small (< 3 GP) vs large clinics (≥ 3 GP) reads − 5.94 (− 11.17; 0.70). It should instead read − 5.94 (− 11.17; − 0.70).
